# Ethics, Poverty and Children’s Vulnerability

**DOI:** 10.1080/17496535.2019.1593480

**Published:** 2019-03-26

**Authors:** Gottfried Schweiger

**Affiliations:** Centre for Ethics and Poverty Research, University of Salzburg, Salzburg, Austria

**Keywords:** Children’s vulnerability, child poverty, child well-being, ethics, responsibilities

## Abstract

This paper is concerned with child poverty from an ethical perspective and applies the normative concept of vulnerability for this purpose. The first part of the paper will briefly outline children’s particular vulnerability and distinguish important aspects of this. Then the concept will be applied to child poverty and it will be shown that child poverty is a corrosive situational vulnerability, with many severe consequences. In this part of the paper normative reasoning and empirical literature will be brought together. Then, the next section will establish why this increased vulnerability of poor children and the harm to their well-being and well-becoming, which they suffer for this reason, are of ethical concern. It will be discussed that child poverty is a structural problem based on social, political and economic factors. The concluding section will then briefly outline why it is imperative to protect children from the vulnerabilities associated with poverty.

## Introduction

Child poverty is a truly global injustice. It affects the lives of billions of children, in developing and developed countries (Batana, Bussolo, and Cockburn 2013; Chzhen et al. 2016). I will not investigate how child poverty should be conceptualised or measured on a global or domestic level, nor will I provide a thorough analysis of all the normative issues involved in child poverty (Schweiger and Graf 2015). Instead I will restrict myself in this paper to exploring one concept, vulnerability, and how it can be used to understand the conceptual and ethical issues involved in child poverty and also to guide obligations towards poor children. Although I will sometimes also mention global child poverty – child poverty in developing countries – my main focus is on child poverty in developed countries and I will mainly rely on knowledge about child poverty in Europe.

## Children’s vulnerability

Vulnerability as a normative concept and in particular children’s vulnerability receives increasing attention in philosophy (Hurst 2015; Straehle 2017; Mackenzie, Rogers, and Dodds 2014b). Here is not the place to discuss the complexity of the issues involved in defining vulnerability and the proposals that have been made to resolve them. I am concerned with the vulnerability of poor children and will only say what is necessary to understand the concept of vulnerability relating to this issue.

Let me begin by introducing the distinction between the inherent and situational vulnerability of children (Mackenzie, Rogers, and Dodds 2014a; Andresen 2014). Using a narrow understanding, inherent vulnerability refers to natural – or ontological – features of children, which define them as children. Examples for this kind of vulnerability are particular developmental needs and the lack of certain abilities (cognitive, physical, emotional). Such inherent vulnerabilities of children make them prone to certain risks and show their inability to protect themselves. Situational vulnerabilities are the vulnerabilities that are based on social practices and norms. For example, the increased vulnerability children face form road traffic. A particular from of situational vulnerability is sometimes called ‘pathogenic’ (Mackenzie, Rogers, and Dodds 2014a), like the vulnerability of girls in a patriarchal society. Inherent and situational vulnerabilities are often intertwined, and situational vulnerabilities are based on, increase and exploit inherent vulnerabilities. A baby’s body is highly vulnerable to physical force (inherent vulnerability) but the fact that some babies are at greater risk of being injured by abuse and corporal punishment is driven by social factors (situational vulnerability). Situational vulnerability can describe a more permanent status (such as in the case of patriarchy), which shapes the lives of particular children. It can also describe changing situations, which all children face more or less frequently (as in the case of road traffic). What is important to add here is that both inherent and situational vulnerability are somehow dynamic features, which change throughout childhood (Schweiger and Graf 2017). As children grow up and acquire abilities, skills and knowledge their inherent and situational vulnerability changes, either because they themselves develop or because the social environment around them treats and views them differently when they are older. Some patriarchal norms and practices might not be so relevant in the lives of female babies, but they become relevant as they become girls and during the transition period on their way to womanhood (Blaise 2009; Kehily 2012). To view children’s vulnerability as a dynamic rather than static feature implies that the vulnerability towards certain dangers decreases, while it increases towards other dangers.

Now I can say something about the relation between vulnerability, harm and ethics in general and specifically in regard to (poor) children’s lives. Vulnerability itself is not necessarily a normative, ethical concept. It can be used descriptively only, though it also has a certain relation to ethics. What I have said so far, and what I will continue to lay out in this and the next section, is both conceptual and descriptive, and I will turn to the ethics aspect after that. In general vulnerability means that a person, a child in this case, is at risk of harm and is unable to sufficiently protect themself or to be sufficiently protected by others (Mackenzie, Rogers, and Dodds 2014a). Vulnerability is in itself not necessarily harmful – although the experience of being vulnerable might be experienced as harmful – but harm enters a child’s life only if the child is vulnerable to that harm. If a child is more vulnerable than another child or adult it can mean different things: it can mean having an increased vulnerability towards a particular harm – describing the likeliness of being harmed in this particular way. Or it can mean being vulnerable to being harmed more severely if the harm occurs – describing the depth of vulnerability. Or it can be understood in the sense of being vulnerable towards more different types of harm – describing the width of vulnerability. So, if a child is more vulnerable this can mean that they are more likely to be harmed (by one or more types of harm), or that the harm will be more severe. Both things can be important from an ethical point of view, but more needs to be known about the particular kinds of harm in question and how they are to be evaluated from an ethical perspective. In general, from a conceptual and ethical perspective, vulnerability is problematic insofar as ethically problematic harm is concerned and, because vulnerability is the channel for harm, they are connected. Now not all kinds of harm are problematic from an ethical point of view, and surely not all kinds of harm are equally problematic. Different ethical theories give different answers about which harm is deemed problematic and why. For example, for an egalitarian the unequal distribution of resources might be harmful, while for sufficientarians this is not always an ethical problem. Also some theories are more concerned with resources and others with capabilities, while a third ethical theory is all about subjective satisfaction. Some types of harm can be justified for a greater benefit like the harm of getting stung by a needle to receive beneficial medical treatment. I will come back to that later in this paper, where I will also say a bit more about harm. Here it is enough to point out that harm and vulnerability are connected and that by exploiting the vulnerability of the child something or someone is causing the harm. Vulnerability is one condition of possibility to be harmed (and to be harmed more severely or more often). This is important for the ethical evaluation of the vulnerability-harm relation and for considering the kind of response that should be sought after. Furthermore, I believe that it is necessary to look both at harms to children’s actual well-being and to harms to their well-becoming (their future well-being). This dual perspective on well-being and well-becoming is of particular interest in the case of children since what happens during childhood has such a crucial influence on the future well-being as an adult, and I will also provide some evidence that growing-up in poverty often has long lasting negative effects.

## Children in poverty

I now want to apply the concept of vulnerability to the situation of children in poverty and explore how it can help us to understand the ethical issues involved here. My approach here is a negative one, which means that I will not start with laying out a theory of justice for children or their moral claims and then apply it to child poverty. In contrast my approach is driven by an examination of child poverty using the lens of vulnerability. What is of ethical concern will emerge during this examination. In particular I want to make four points about the relationship between vulnerability and child poverty.

Firstly, children in poverty are children and as such are in need of protection. That seems obvious, but this is the first dimension of inherent and situational vulnerability, and we should not forget about it. That has three implications: firstly, they are a quite diverse group of beings, ranging from newborns to teenagers, and their inherent and situational vulnerabilities vary accordingly. So, if I paint the picture here of children’s vulnerability in poverty, I am forced to do so with a broad brush, which cannot do justice to the differences based on development levels, abilities and characteristics of different age groups. Secondly, children’s vulnerability is in general greater than that of adults (exceptions exist), and childhood is a particularly sensitive phase of life, which shapes future outcomes and prospects (Duncan et al. 2012). Thirdly, children are in need of care and protection and thus they are highly dependent on other people and institutional settings (for example schools, health care) to meet their needs (C. Macleod 2015; Mullin 2014a). The particular situational vulnerability of child poverty is on top of their general vulnerability and influences it, as well as their chances of experiencing harm.

Secondly, poverty is a situational vulnerability in children’s lives. It is not an inherent feature of some children but is rather a social phenomenon. That comes in my opinion with several important implications, some of them not uncontroversial. Child poverty is multi-dimensional in that it is not only about money (or the income of the parents) but about multiple deprivations in many areas of a child’s life (Biggeri, Trani, and Mauro 2010; Boyden and Bourdillon 2012). For the purpose of this paper, it is not important whether that deprivation is conceptualised and measured using capabilities, goods, needs or rights (Minujin et al. 2006; Bastos and Machado 2009; Alkire and Roche 2012). The research evidence is convincing that poverty during childhood affects a wide range of goods (e.g. food, clothes, housing, sanitation) (Ridge 2011; UNICEF Innocenti Research Centre 2012; Boyden and Bourdillon 2012) and that these deprivations can be and often are translated into physical, psychological, behavioural and social harm (e.g. lower health, depression, isolation, delinquency) (Ridge 2011; Evans and Cassells 2014; Ferguson, Bovaird, and Mueller 2007; Yoshikawa, Lawrence Aber, and Beardslee 2012). These are impressive examples of how situational and inherent vulnerabilities intertwine and how social factors exploit inherent vulnerabilities (children’s bodies and minds). Furthermore, the situational vulnerability of child poverty does not affect all children (only those who are poor, obviously) and it is not the same for all poor children. This means that the situational vulnerability of poor children is further mediated (enhanced or diminished) by several other factors, which in turn can be understood as situational or inherent vulnerabilities if they are vulnerability-enhancers. On the one hand, examples for vulnerability-enhancers are disability or innate health issues (as a form of inherent vulnerability) or racial discrimination (as a form of situational vulnerability) (Shahtahmasebi et al. 2011; Phung 2008). Vulnerability diminishers, on the other hand, are for example strong social networks, well established social services, or innate psychological resilience factors (Felner and DeVries 2013). Growing-up in poverty is not necessarily harmful for all children, and many of them develop well and are able to escape poverty in later life. These exceptions – and they are exceptions both on the domestic and the global level – do not speak against my claim that poverty makes children more vulnerable and that this vulnerability in most cases translates into some harms and disadvantages.

Thirdly, poverty is that kind of situational vulnerability, which affects other situational vulnerabilities. Borrowing a concept from Jonathan Wolff and Avner de-Shalit (Wolff and de-Shalit 2007), poverty can be understood as a corrosive disadvantage, or, to stay in the conceptual language of this paper, a corrosive vulnerability. What is meant by that is that being poor affects the spaces and times of vulnerability where and within which children grow up, and that determines to which (additional) threats they become vulnerable. I mentioned earlier such situational vulnerabilities as the exposure to road traffic. In poor children’s lives such risks and dangers can be more often present, or they are less protected and guarded from them. The corrosiveness can then lead to an accumulation of situational vulnerabilities. Examples, which I take from the empirical literature on child poverty are: the exposure to toxic environments (Wakefield and Baxter 2010); to live in neighbourhoods with high crime rates (Livingston, Kearns, and Bannister 2014); to grow up in families which experience all kinds of different problems (domestic violence, corporal punishment, alcoholism, drug use), (Goodman et al. 2009; Hooper et al. 2007; Wadsworth 2012); early contact with drugs (Karriker-Jaffe 2011), and anti-social behaviour by their peers (Zimmerman and Messner 2011); having to go to less well-equipped schools and having overburdened teachers (Lupton 2005; Auwarter and Aruguete 2008), or being alone outside more often. These are some examples of situational vulnerabilities and poor children are exposed to these dangers and risks more often than their non-poor peers, and for unjust reasons.

Fourthly, poverty has a temporal dimension. On the one hand, poverty can be short-term or long-term, or even chronic. It can be a defining feature of a whole childhood (and life) or it can be a (temporary) situation that comes and goes while a child grows up. On the other hand, poverty can have short-term and long-term effects. This is connected to the duration of poverty but can be quite detached because shorter periods of (severe) poverty can have long-lasting effects. Research points in the direction of child poverty being only properly understood if it is seen in this light as (potentially) affecting the whole course of the child’s life, something which makes the child vulnerable to a wide range of negative effects in later life as well. Growing up in poverty makes children vulnerable to embarking on a path of delinquency (Rekker et al. 2015), lower educational achievements (McKinney 2014), early pregnancy (Conrad 2012), health problems in later life (Conroy, Sandel, and Zuckerman 2010; Raphael 2011) as well as adult unemployment and poverty (van Ham et al. 2014; Mentis 2015), which I have already mentioned. Periods of poverty have a negative impact on life expectancy and quality-adjusted life years (Huisman et al. 2013; Duncan et al. 2012). The pathways of how the situational vulnerability of poverty spills over to so many other areas of life and creates vulnerabilities, sometimes even years or decades after childhood, are not currently fully understood. It is likely that a combination of physical, psychological, and social factors comes together here (McEwen and McEwen 2017), and that early poverty can have a significant influence on brain development (Kim et al. 2013; Balter 2015).

## The ethical significance of poor children’s vulnerability

So far the work I have done in this paper was descriptive and analytical but it touched on many normative issues. But children’s vulnerability is not always of particular ethical concern and some inherent and situational vulnerabilities are morally neutral, or even beneficial. Two aspects are of ethical concern though: that some children are more vulnerable than others for unjustified reasons, and that some children are less protected from harm than others. Harm and vulnerabilities are connected, as I mentioned in the first section of this paper, in that harm exploits vulnerabilities. Both are highly relevant for child poverty.

Now, on the one hand, child poverty, as I showed in the section before, creates several new situational vulnerabilities in children’s lives, vulnerabilities which can finally translate into harm towards the physical, psychological and social well-being and well-becoming of children (Schweiger 2015). On the other hand, as research also points out, children in poverty suffer several poverty and non-poverty related kinds of harm (or have a greater risk to suffer from them), because they are not sufficiently protected. With poverty related harm I mean such harm that is inherent in the nature of poverty. This harm is the direct result of the situational vulnerability of poverty. Examples for such harm include the lack of poverty-defining goods such adequate clothes, housing, food or toys (Ridge 2011; UNICEF Innocenti Research Centre 2012). Non-poverty related harm is harm which is not inherent in the poverty situation of the child – it does not define poverty as poverty – but which can also have causes other than poverty. The situational vulnerability of poverty, and the situational vulnerabilities it causes through its corrosiveness, makes it more likely that children would suffer from this harm and poverty can play a causal role in its occurrence, but that is not defining poverty nor is it necessary for the poverty situation of a child. Examples for such non-poverty related harm are health issues, behavioural problems, the experience of abuse and violence, or educational shortcomings and later unemployment.

For these issues to be a matter of justice, it needs to be established that the creation of these situational vulnerabilities and the actual occurrence of associated harm violate the moral claims of children and are thus unjustified (Schweiger and Graf 2015). As I said in the beginning of this paper my approach here does not apply a fleshed-out concept of justice for children, or a fully developed ethical theory of their moral claims. Rather my approach is a negative and partial one, which started with an examination of the issues involved in child poverty through a conceptual analysis of vulnerability. The issues of children’s increased vulnerability and how children in poverty are endangered in their well-being and well-becoming (Graf and Schweiger 2015) compared to their non-poor peers emerged through this. Both the inequality between poor and non-poor children and the increased vulnerability and experience of several non-trivial types of harm through poverty signal an ethical problem and are in need of justification. Otherwise they are unjust. I suppose that many different ethical theories would converge on this conclusion, such as both a sufficientarian and an egalitarian account of justice for children (Macleod 2010). While sufficientarianism would have no quarrel with some inequality between children (that some children are more vulnerable than others) as long as no child is too vulnerable, egalitarianism would assume roughly equal vulnerability of all children as just (assuming a certain level of vulnerability as just). So from the standpoint of sufficientarianism it is necessary to establish a threshold of well-being and well-becoming, to which all children are entitled to as a matter of justice (Schweiger and Graf 2015). It would go beyond the scope of this paper to do that in detail but I believe that the evidence about the relation of vulnerability, harm and child poverty, which I discussed in the previous section, at least suggests, if not proves that child poverty harms children’s well-being and well-becoming to such a degree that these children fall short of what they are entitled to as a matter of justice. Likewise I assume that egalitarians would assume that all children are indeed entitled to such a level of well-being and well-becoming, which is higher than those of children in poverty. I used a positive phrasing in the last sentences but the point can also be made in the conceptual language of vulnerability and harm. Sufficientarianism needs to establish a threshold of how much vulnerability and harm is ethically unproblematic, and I believe that the exercise of exploring the degree to which poor children are actually vulnerable and harmed, gives sufficient reasons to believe that they are too vulnerable and experience too much harm.

It is now the task of the rest of this paper to establish that this increased vulnerability and the harm associated with child poverty are indeed of ethical concern because they are unjustified. Furthermore, in the case of child poverty, it is important to treat it as a social phenomenon and not an individual case, and thus I am not concerned with individual cases but with the population of poor children. This shifts the attention, where it should be, onto the structural causes and factors which create and sustain poverty, both in developed and developing countries.

Now, plausible reasons that could justify some children being more vulnerable than others seem to comprise natural and unpreventable causes (such as a genetic disease); children’s free and autonomous choices (which are only relevant for older and adult-like mature children); if tragic choices need to be made (for example in the case of organ transplantations if not enough organs are available), or if the children themselves or someone else will benefit significantly in the long-run (for example a risky surgical procedure or an organ donation). All of these cases can be tricky, and demand further scrutiny in some cases (for example, it needs to be established how to calculate the gains and losses of vulnerability). For the issue considered in this paper I am confident that the evidence is sufficient that such detailed analysis is not needed to reach a robust conclusion regarding the ethical status of child poverty as an unjust vulnerability and harm.

Child poverty is certainly not a natural condition but a social condition. Children are born into poverty or become poor not in the same way as they contract a disease or inherent their genome, but by social causes. It is worth noting that low health and disabilities can contribute to becoming poor or staying poor under such social conditions, where there is no sufficient (private or public) support, and social norms and practices can attach lower social status, class and other disadvantages to all different kinds of natural features and characteristics like sex, race or ethnicity. The social causes behind such inequalities are what need to be addressed and critically evaluated. Child poverty shows the following characteristics: poverty is a widespread phenomenon in all societies, but is particularly severe in developing countries. It is the result of the maldistribution of income and other resources between the members of a society, and between countries. In developed countries in particular enough income and other resources and assets are available that no one would need to be poor, and that is also not impossible on a global level. So, while poverty and inequality are closely tied, they are not the same. As Amartya Sen (Sen 1985) has rightly pointed out, there is what he calls an ‘absolute core’ of poverty, which is different from inequality. It is possible that inequalities in income and other poverty defining goods exist, without there being any poverty. The opposite seems implausible: a world in which all are poor.

The underlying causes for child poverty are hard to disentangle, but I want to name some of the most important ones: economic inequalities based on unemployment and low wages combined with a lack of social welfare, which could compensate for this lack. In the background there are, in developed countries, structures of profit-maximisation, the penalisation of poverty (Wacquant 2009); unequal access to and chances in the labour market competition and the extension of precarious work (Standing 2016; Kalleberg 2011; Chzhen 2017); restructuring of the welfare system (Lødemel and Trickey 2001; Schram et al. 2009); the individualisation of responsibility for one’s welfare and market success (Honneth 2004); international and global changes in value chains (Levy 2005); competition between countries (based on their labour force, tax systems, natural resources etc.), which pressures them to lower welfare standards and to deregulate labour standards (Hermann 2014; Shin 2000), and, finally, the lack of fair tax systems, which allow rich individuals and corporations to avoid taxation and to maximise their labour-free incomes based on financial and other assets (Sayer 2015). Obviously, nearly all of these factors are not directly related to children and their situation but rather target and pressure adults (as individuals and families), and their precarious situation then spills over to their children. But there are also important groups of children in poverty (in developed countries), who suffer directly: children in foster care, unaccompanied minor refugees, street children without a (functioning) family background and older children who have assumed responsibility for themselves. For all these structural factors and background conditions (one might call them structural injustices (Young 2011)) children are not responsible and they are in a particularly weak position to change them or to improve their situation. On a global scale, the birth privilege is immense, and also within developed countries life chances are largely determined by the parents (but state institutions can do a lot to improve chances and to reach more equality of opportunity).

It is also highly implausible to attribute any responsibility to the children themselves for their poverty. On the one hand, childhood is a phase that deserves special protection and that influences if, when and how children should be held responsible for their actions. Younger children lack the abilities, knowledge and skills to make rational choices for themselves and they are in need of guidance in many aspects of their lives. But autonomy and rationality are preconditions to being held responsible. Both develop during childhood, and there are valid reasons to treat children differently also in respect to those choices which they make and in those areas of their lives where they are competent to make these choices. Competence is not the single important benchmark here, but is a reason to let children safely experiment and to provide them with more room to make mistakes, and to learn from those mistakes that there are also other important aspects of the process of growing-up and becoming autonomous (Anderson and Claassen 2012; Franklin-Hall 2013). On the other hand, the structural background conditions, mentioned above, which cause and sustain (child) poverty are well out of the reach of children, whether or not they are competent in some areas of their lives. Children are not in a position to alter them effectively, because they are born into them and growing up poor affects them, their abilities, knowledge and skills in so many ways. This does not mean that all poor children are completely helpless – they are when they are very young – and that they do not comprehend their situation. Children are often well aware of their own social status and that of their family and they often develop ways to cope and deal with their situation with the aim to improve it. The phenomenon of ‘young carers’ is one of many examples that shows how skilful, resourceful and resilient children can be when they face adversity (Rose and Cohen 2010). Others choose a path of aggression, violence and delinquency, which can severely damage their life chances (Rekker et al. 2015).

Regarding the question of whether child poverty might also have some benefits for the children themselves or others it seems necessary to mention three points. Firstly, children in poverty do not profit either short-term or long term from growing up poor; it is, as shown throughout this paper, the other way around. Growing up poor is often a lifetime disadvantage and increases vulnerability throughout the course of life. Secondly, it is hard to think of any other agent to profit from child poverty. Their parents certainly do not, and also for communities and the state child poverty is costly (Holzer et al. 2008; Attree 2006). Though, thirdly, it is not as if no one profits from child poverty (Hatcher 2016), directly and indirectly. Children in poverty are a cheap labour force, they are exploited globally, used as organ donors, criminalised and penalised and put in privatised for-profit institutions, and they can be used as scapegoats to justify social control over them and their families. It is not hard to see that these kinds of profit are not justifiable as reasons to produce or sustain child poverty, because they do not benefit anyone worse-off than the children in poverty, the benefits achieved for others are certainly not of such ethical importance (it is overwhelmingly a case of dirty money made by people who could earn their living in other ways), and the children, and their care givers, have either no real opportunity to choose against being exploited.

Lastly, it is important to point out that I approach this from a child-centred perspective, which is not about the parents or the family as the unit of concern. This has two implications. Firstly, parents might play a role in their children (and themselves) being poor, which deserves blame. They could have made bad decisions or leave their children deprived although they could do better. I suppose that this is not very often the case, and that in the vast majority of cases, the parents are not to be held responsible for themselves or their children being poor. Rather, research shows that parents make all kind of sacrifices to protect their children from poverty and to provide for them as well as possible (Main and Bradshaw 2016; Russell, Harris, and Gockel 2008). In this respect, I do not think that a family-centred analysis would come to significantly different results. Still, I believe it is important to focus on the child and her well-being (and vulnerability and harm), and to respect her in her own right. Secondly, that said, the family is certainly a very important mediator and facilitator. The family can protect children in poverty from many poverty-related and non-poverty-related types of harm and reduce the situational vulnerability significantly. Likewise, the family is an important place, where vulnerabilities and harm are created and sustained. The actions of the parents, and siblings and other family members, can be severely harmful and dangerous, and families can become spaces of extensive vulnerability, for example when domestic violence and abuse are frequent and family life is overshadowed by an atmosphere of fear, broken promises, humiliation or aggression.

What I tried to establish in this section, was that child poverty, by creating vulnerabilities and making children more likely to be harmed and to be less well protected against them (compared with other children), is of ethical concern and this establishes it as an injustice. Child poverty is unjust because it makes children more vulnerable, and more often harmed, for unjustified reasons since today’s societies are shaped by social, cultural, political, and most importantly economic factors and background conditions beyond the reach of children and their families.

## Conclusion: responding to child poverty

In the concluding section of this paper I will now turn my attention to ethical responses to child poverty. Four points are important here. Firstly, although there is very good research on child poverty and what it does to children, there is much less research on the main causes for (child) poverty at a country and global level and the causation is less well understood. To research the lives of children and their families is, it seems, easier than to find out why they are in this position beyond stating individual characteristics and factors (low income, unemployment, health issues, single mothers), which do not uncover the underlying causes. Rather, I would want to say, research on such individual risk factors is concerned with symptoms and not the real underlying problems and their causes (O’Connor 2001).

Secondly, if child poverty is such a threat to children’s well-being and well-becoming, as I have shown in this paper so far, then it is imperative to safeguard children and to protect them. But if the underlying causes for their poverty are not well understood it appears that the responses to child poverty are also merely treating symptoms. Or, to put it differently, the causes of child poverty should be eradicated, but for that structural changes are needed.

Thirdly, if that is not possible, out of reach or simply not on the political agenda, then it still remains an ethical imperative to do as much as possible to protect poor children, to enable them to escape their poverty and to protect them from the many kinds of harm which could follow from their situational vulnerability of being poor. That is where the huge arsenal of social policy and social work enters the lives of poor children and their families. Policies and social work can have a huge positive impact but, unfortunately, as I have already mentioned, they are under attack and policies are restructured to serve neoliberal agendas (Jensen and Tyler 2015; Crossley 2016). Such restructuring reduces the effectiveness of services for poor children and instead, unjustifiably but powerfully, blames their parents (Simpson, Lumsden, and McDowall Clark 2015).

And, fourthly, social policies and social work are themselves pervaded by ethical issues. I want to mention a few of them to conclude this paper: The effects of policies and methods of working with children are sensitive, and there is always a distinct possibility they enhance the vulnerabilities or inflict harm on some children even if they achieve the opposite in other children. Differential outcomes based on several factors (age, gender, family structure, health, disability, race) must be dealt with if justice for all children is the desired goal. Then, the state and its institutions as well as social workers and other professionals are often in a position of power in contrast to children and their families. There is a distinct danger of abuse in such relations of inequality and dependency, and experiences of humiliation and infantilising are common. This is also connected to issues of paternalism and tutelage. While there is room for paternalism towards children *qua* them being children (Mullin 2014b), and also because they are vulnerable and harmed if they are poor, paternalism is neither always justified nor should it be applied without sensitivity and respect. And, most often it is not the child who is targeted but their parents and care-givers. Finally, under circumstances of scarce (material and human) resources it is necessary to prioritise some children and families over others based on differential evaluation of their vulnerabilities and potential types of harm (Dixon and Nussbaum 2012). Such decisions face several difficulties, some of which are epistemic (what is and can be known about the child and its situation and about the likely effects to be expected), while others are ethical (how should vulnerabilities and harm be ranked and what conclusions are to be drawn from the empirical evidence available) or political (how do certain policies spill over into other policy areas, what ideological assumptions lie behind certain policies).

The framework of vulnerability laid out here might help to navigate and resolve some of these ethical issues, for example by putting the priority on corrosive vulnerabilities and those children who are the most vulnerable. But that needs to be left to another occasion.

## References

[CIT0001] Alkire Sabina, and Roche José Manuel 2012 “Beyond Headcount: Measures That Reflect the Breadth and Components of Child Poverty.” In *Global Child Poverty and Well-Being*. 1st ed, edited by Minujin Alberto and Nandy Shailen, 103–133. Bristol: Policy Press.

[CIT0002] Anderson Joel, and Claassen Rutger 2012 “Sailing Alone: Teenage Autonomy and Regimes of Childhood.” *Law and Philosophy* 31 (5): 495–522. doi:10.1007/s10982-012-9130-9.

[CIT0003] Andresen Sabine. 2014 “Childhood Vulnerability: Systematic, Structural, and Individual Dimensions.” *Child Indicators Research* 7 (4): 699–713. doi:10.1007/s12187-014-9248-4.

[CIT0005] Attree Pamela. 2006 “The Social Costs of Child Poverty: A Systematic Review of the Qualitative Evidence.” *Children & Society* 20 (1): 54–66. doi:10.1002/chi.854.

[CIT0006] Auwarter Amy E., and Aruguete Mara S. 2008 “Effects of Student Gender and Socioeconomic Status on Teacher Perceptions.” *The Journal of Educational Research* 101 (4): 242–246. doi:10.3200/JOER.101.4.243-246.

[CIT0007] Balter Michael. 2015 “Poverty May Affect the Growth of Children’s Brains.” *Science*, doi:10.1126/science.aab0395.

[CIT0008] Bastos Amélia, and Machado Carla 2009 “Child Poverty: A Multidimensional Measurement.” *International Journal of Social Economics* 36 (3): 237–251. doi:10.1108/03068290910932738.

[CIT0009] Batana Yélé, Bussolo Maurizio, and Cockburn John 2013 “Global Extreme Poverty Rates for Children, Adults and the Elderly.” *Economics Letters* 120 (3): 405–407. doi:10.1016/j.econlet.2013.05.006.

[CIT0010] Biggeri Mario, Trani Jean-Francois, and Mauro Vincenzo 2010 *The Multidimensionality of Child Poverty: An Empirical Investigation on Children of Afghanistan.* OPHI Research in Progress Oxford: Oxford Poverty & Human Development Initiative http://www.ophi.org.uk/wp-content/uploads/OPHI-RP19a.pdf.

[CIT0011] Blaise Mindy. 2009 “‘What a Girl Wants, What a Girl Needs’: Responding to Sex, Gender, and Sexuality in the Early Childhood Classroom.” *Journal of Research in Childhood Education* 23 (4): 450–460. doi:10.1080/02568540909594673.

[CIT0012] Boyden Jo, and Bourdillon Michael 2012 *Childhood Poverty: Multidisciplinary Approaches*. 1st ed Basingstoke: Palgrave Macmillan.

[CIT0013] Chzhen Yekaterina. 2017 “Unemployment, Social Protection Spending and Child Poverty in the European Union During the Great Recession.” *Journal of European Social Policy* 27 (2): 123–137. doi:10.1177/0958928716676549.

[CIT0014] Chzhen Yekaterina, de Neubourg Chris, Plavgo Ilze, and de Milliano Marlous 2016 “Child Poverty in the European Union: The Multiple Overlapping Deprivation Analysis Approach (EU-MODA).” *Child Indicators Research* 9 (2): 335–356. doi:10.1007/s12187-015-9321-7.

[CIT0015] Conrad David. 2012 “Deprivation-Based Inequalities in Under-18 Conception Rates and the Proportion of Under-18 Conceptions Leading to Abortion in England, 1998–2010.” *Journal of Public Health* 34 (4): 609–614. doi:10.1093/pubmed/fds031. 22615419

[CIT0016] Conroy Kathleen, Sandel Megan, and Zuckerman Barry 2010 “Poverty Grown Up: How Childhood Socioeconomic Status Impacts Adult Health.” *Journal of Developmental & Behavioral Pediatrics* 31 (2): 154–160. doi:10.1097/DBP.0b013e3181c21a1b. 20145476

[CIT0017] Crossley Stephen. 2016 “‘Realising the (Troubled) Family’, ‘crafting the Neoliberal State’.” *Families, Relationships and Societies* 5 (2): 263–279. doi:10.1332/204674315X14326465757666.

[CIT0018] Dixon Rosalind, and Nussbaum Martha 2012 “Children’s Rights and a Capabilities Approach: The Question of Special Priority.” *Cornell Law Review* 97: 549–593.

[CIT0019] Duncan Greg J., Magnuson Katherine, Kalil Ariel, and Ziol-Guest Kathleen 2012 “The Importance of Early Childhood Poverty.” *Social Indicators Research* 108 (1): 87–98. doi:10.1007/s11205-011-9867-9.

[CIT0020] Evans Gary W., and Cassells Rochelle C. 2014 “Childhood Poverty, Cumulative Risk Exposure, and Mental Health in Emerging Adults.” *Clinical Psychological Science* 2 (3): 287–296. doi:10.1177/2167702613501496. 26609499 PMC4655888

[CIT0021] Felner Robert D., and DeVries Melissa L. 2013 “Poverty in Childhood and Adolescence: A Transactional–Ecological Approach to Understanding and Enhancing Resilience in Contexts of Disadvantage and Developmental Risk.” In *Handbook of Resilience in Children*. 1st ed, edited by Goldstein Sam, and Brooks Robert B., 105–126. Boston, MA: Springer http://www.springerlink.com/index/10.1007/978-1-4614-3661-4_7.

[CIT0022] Ferguson Hb, Bovaird S., and Mueller Mp 2007 “The Impact of Poverty on Educational Outcomes for Children.” *Paediatrics & Child Health* 12 (8): 701–706. 19030450 10.1093/pch/12.8.701 PMC2528798

[CIT0023] Franklin-Hall Andrew. 2013 “On Becoming an Adult: Autonomy and the Moral Relevance of Life’s Stages.” *The Philosophical Quarterly* 63 (251): 223–247. doi:10.1111/1467-9213.12014.

[CIT0024] Goodman Lisa A, Smyth Katya Fels, Borges Angela M, and Singer Rachel 2009 “When Crises Collide” *Trauma, Violence & Abuse* 10 (4): 306–329. doi:10.1177/1524838009339754. 19776085

[CIT0025] Graf Gunter, and Schweiger Gottfried eds. 2015 *The Well-Being of Children: Philosophical and Social Scientific Approaches*. 1st ed Berlin: DeGruyter Open.

[CIT0026] Hatcher Daniel L. 2016 *The Poverty Industry: The Exploitation of America’s Most Vulnerable Citizens. 1st ed. Families, Law, and Society Series*. New York: New York University Press.

[CIT0027] Hermann Christoph. 2014 “Structural Adjustment and Neoliberal Convergence in Labour Markets and Welfare: The Impact of the Crisis and Austerity Measures on European Economic and Social Models.” *Competition & Change* 18 (2): 111–130. doi:10.1179/1024529414Z.00000000051.

[CIT0028] Holzer Harry J., Schanzenbach Diane Whitmore, Duncan Greg J., and Ludwig Jens 2008 “The Economic Costs of Childhood Poverty in the United States.” *Journal of Children and Poverty* 14 (1): 41–61. doi:10.1080/10796120701871280.

[CIT0029] Honneth Axel. 2004 “Organized Self-Realization.” *European Journal of Social Theory* 7 (4): 463–478. doi:10.1177/1368431004046703.

[CIT0030] Hooper Carol-Ann, Gorin Sarah, Cabral Christie, and Dyson Claire 2007 *Living with Hardship 24/7: The Diverse Experiences of Families in Poverty in England*. 1st ed London: Frank Buttle Trust http://www.york.ac.uk/admin/presspr/features/hardship.pdf.

[CIT0031] Huisman Martijn, Read Sanna, Towriss Catriona A., Deeg Dorly J. H., and Grundy Emily 2013 “Socioeconomic Inequalities in Mortality Rates in Old Age in the World Health Organization Europe Region.” *Epidemiologic Reviews* 35 (1): 84–97. doi:10.1093/epirev/mxs010. 23382476

[CIT0032] Hurst Samia. 2015 “Clarifying Vulnerability: The Case of Children.” *Asian Bioethics Review* 7 (2): 126–138. doi:10.1353/asb.2015.0018.

[CIT0033] Jensen Tracey, and Tyler Imogen 2015 “‘Benefits Broods’: The Cultural and Political Crafting of Anti-Welfare Commonsense.” *Critical Social Policy* 35 (4): 470–491. doi:10.1177/0261018315600835.

[CIT0034] Kalleberg Arne L. 2011 *Good Jobs, Bad Jobs : The Rise of Polarized and Precarious Employment Systems in the United States, 1970s to 2000s*. 1st ed New York: Russell Sage Foundation.

[CIT0035] Karriker-Jaffe Katherine J. 2011 “Areas of Disadvantage: A Systematic Review of Effects of Area-Level Socioeconomic Status on Substance use Outcomes.” *Drug and Alcohol Review* 30 (1): 84–95. doi:10.1111/j.1465-3362.2010.00191.x. 21219502 PMC3057656

[CIT0036] Kehily Mary Jane. 2012 “Contextualising the Sexualisation of Girls Debate: Innocence, Experience and Young Female Sexuality.” *Gender and Education* 24 (3): 255–268. doi:10.1080/09540253.2012.670391.

[CIT0037] Kim Pilyoung, Evans Gary W., Angstadt Michael, Shaun Ho S., Sripada Chandra S., Swain James E., Liberzon Israel, and Luan Phan K. 2013 “Effects of Childhood Poverty and Chronic Stress on Emotion Regulatory Brain Function in Adulthood.” *Proceedings of the National Academy of Sciences*, October, 1–6. doi:10.1073/pnas.1308240110. PMC3831978 24145409

[CIT0038] Levy David L. 2005 “Offshoring in the New Global Political Economy.” *Journal of Management Studies* 42 (3): 685–693. doi:10.1111/j.1467-6486.2005.00514.x.

[CIT0039] Livingston Mark, Kearns Ade, and Bannister Jon 2014 “Neighbourhood Structures and Crime: The Influence of Tenure Mix and Other Structural Factors upon Local Crime Rates.” *Housing Studies* 29 (1): 1–25. doi:10.1080/02673037.2014.848267.

[CIT0040] Lødemel Ivar, and Trickey Heather eds. 2001 *‘An Offer You Can’t Refuse’: Workfare in International Perspective*. 1st ed Bristol: Policy Press.

[CIT0041] Lupton Ruth. 2005 “Social Justice and School Improvement: Improving the Quality of Schooling in the Poorest Neighbourhoods.” *British Educational Research Journal* 31 (5): 589–604. doi:10.1080/01411920500240759.

[CIT0042] Mackenzie Catriona, Rogers Wendy, and Dodds Susan 2014a “Introduction: What Is Vulnerability and Why Does It Matter for Moral Theory?” In *Vulnerability: New Essays in Ethics and Feminist Philosophy*, edited by Mackenzie Catriona, Rogers Wendy, and Dodds Susan, 1–29. New York: Oxford University Press.

[CIT0043] Mackenzie Catriona, Rogers Wendy, and Dodds Susan 2014b *Vulnerability: New Essays in Ethics and Feminist Philosophy*. 1st ed Studies in Feminist Philosophy New York: Oxford University Press.

[CIT0044] Macleod Colin M. 2010 “Primary Goods, Capabilities and Children.” In *Measuring Justice – Primary Goods and Capabilities*, edited by Brighouse Harry, and Robeyns Ingrid, 174–192. Cambridge: Cambridge University Press.

[CIT0045] Macleod Colin. 2015 “Agency, Authority and the Vulnerability of Children.” In *The Nature of Children’s Well-Being*, Vol. 9. 1st ed, edited by Bagattini Alexander, and Macleod Colin, 53–64. Dordrecht: Springer http://link.springer.com/10.1007/978-94-017-9252-3_4.

[CIT0046] Main Gill, and Bradshaw Jonathan 2016 “Child Poverty in the UK: Measures, Prevalence and Intra-Household Sharing.” *Critical Social Policy* 36 (1): 38–61. doi:10.1177/0261018315602627.

[CIT0047] McEwen Craig A., and McEwen Bruce S. 2017 “Social Structure, Adversity, Toxic Stress, and Intergenerational Poverty: An Early Childhood Model.” *Annual Review of Sociology* 43 (1): 445–472. doi:10.1146/annurev-soc-060116-053252.

[CIT0048] McKinney Stephen. 2014 “The Relationship of Child Poverty to School Education.” *Improving Schools* 17 (3): 203–216. doi:10.1177/1365480214553742.

[CIT0049] Mentis Alexios-Fotios A. 2015 “To What Extent Are Greek Children Exposed to the Risk of a Lifelong, Intergenerationally Transmitted Poverty?.” *Poverty & Public Policy* 7 (4): 357–381. doi:10.1002/pop4.123.

[CIT0050] Minujin Alberto, Delamonica Enrique, Davidziuk Alejandra, and Gonzalez Edward D 2006 “The Definition of Child Poverty: A Discussion of Concepts and Measurements.” *Environment and Urbanization* 18 (2): 481–500. doi:10.1177/0956247806069627.

[CIT0051] Mullin Amy. 2014a “Children, Vulnerability, and Emotional Harm.” In *Vulnerability*. 1st ed, edited by Mackenzie Catriona, Rogers Wendy, and Dodds Susan, 266–287. New York: Oxford University Press.

[CIT0052] Mullin Amy. 2014b “Children, Paternalism and the Development of Autonomy.” *Ethical Theory and Moral Practice* 17 (3): 413–426. doi:10.1007/s10677-013-9453-0.

[CIT0053] O’Connor Alice. 2001 *Poverty Knowledge : Social Science, Social Policy, and the Poor in Twentieth-Century U.S. History*. 1st ed Princeton, NJ: Princeton University Press.

[CIT0054] Phung Viet-Hai. 2008 “Ethnicity and Child Poverty Under New Labour: A Research Review.” *Social Policy and Society* 7 (04), doi:10.1017/S1474746408004491.

[CIT0055] Raphael Dennis. 2011 “Poverty in Childhood and Adverse Health Outcomes in Adulthood.” *Maturitas* 69 (1): 22–26. doi:10.1016/j.maturitas.2011.02.011. 21398059

[CIT0056] Rekker Roderik, Pardini Dustin, Keijsers Loes, Branje Susan, Loeber Rolf, and Meeus Wim 2015 “Moving in and out of Poverty: The Within-Individual Association Between Socioeconomic Status and Juvenile Delinquency.” Edited by Karin Bammann *PLOS ONE* 10 (11): e0136461. doi:10.1371/journal.pone.0136461. 26575271 PMC4648521

[CIT0057] Ridge Tess. 2011 “The Everyday Costs of Poverty in Childhood: A Review of Qualitative Research Exploring the Lives and Experiences of Low-Income Children in the UK.” *Children & Society* 25 (1): 73–84. doi:10.1111/j.1099-0860.2010.00345.x.

[CIT0058] Rose Helena D., and Cohen Keren 2010 “The Experiences of Young Carers: A Meta-Synthesis of Qualitative Findings.” *Journal of Youth Studies* 13 (4): 473–487. doi:10.1080/13676261003801739.

[CIT0059] Russell Mary, Harris Barbara, and Gockel Annemarie 2008 “Parenting in Poverty: Perspectives of High-Risk Parents.” *Journal of Children and Poverty* 14 (1): 83–98. doi:10.1080/10796120701871322.

[CIT0101] Sayer Andrew. 2015 *Why We Can’t Afford the Rich*. 1st ed Bristol: The Policy Press.

[CIT0060] Schram Sanford F, Soss Joe, Fording Richard, and Houser Linda 2009 “Deciding to Discipline: Race, Choice, and Punishment at the Frontlines of Welfare Reform.” *American Sociological Review* 74 (3): 398–422. doi:10.1177/000312240907400304.

[CIT0061] Schweiger Gottfried. 2015 “Justice and Children’s Well-Being and Well-Becoming.” In *The Well-Being of Children: Philosophical and Social Scientific Approaches*. 1st ed, edited by Graf Gunter, and Schweiger Gottfried, 84–96. Berlin: DeGruyter Open.

[CIT0062] Schweiger Gottfried, and Graf Gunter 2015 *A Philosophical Examination of Social Justice and Child Poverty*. 1st ed Basingstoke: Palgrave Macmillan http://www.palgraveconnect.com/doifinder/10.1057/9781137426024.

[CIT0063] Schweiger Gottfried, and Graf Gunter 2017 “Ethics and the Dynamic Vulnerability of Children.” *Les Ateliers de l’éthique/The Ethics Forum* 12 (2–3): 243–261. doi:10.7202/1051284ar.

[CIT0064] Sen Amartya. 1985 “A Sociological Approach to the Measurement of Poverty: A Reply to Professor Peter Townsend.” *Oxford Economic Papers* 37 (4): 669–676.

[CIT0065] Shahtahmasebi Said, Emerson Eric, Berridge Damon, and Lancaster Gillian 2011 “Child Disability and the Dynamics of Family Poverty, Hardship and Financial Strain: Evidence From the UK.” *Journal of Social Policy* 40 (04): 653–673. doi:10.1017/S0047279410000905.

[CIT0066] Shin Dong-Myeon. 2000 “Economic Policy and Social Policy: Policy-Linkages in an Era of Globalisation.” *International Journal of Social Welfare* 9 (1): 17–30. doi:10.1111/1468-2397.00105.

[CIT0067] Simpson Donald, Lumsden Eunice, and McDowall Clark Rory 2015 “Neoliberalism, Global Poverty Policy and Early Childhood Education and Care: A Critique of Local Uptake in England.” *Early Years* 35 (1): 96–109. doi:10.1080/09575146.2014.969199.

[CIT0068] Standing Guy. 2016 *The Precariat: The New Dangerous Class*. Revised edition London: Bloomsbury Academic.

[CIT0069] Straehle Christine. 2017 *Vulnerability, Autonomy, and Applied Ethics*. New York: Routledge http://www.tandfebooks.com/isbn/9781315647418.

[CIT0070] UNICEF Innocenti Research Centre 2012 *Measuring Child Poverty: New League Tables of Child Poverty in the World’s Rich Countries. Innocenti Report Card 10*. Florence: UNICEF Innocenti Research Centre http://www.unicef.gr/pdfs/RC10_report.pdf.

[CIT0071] van Ham Maarten, Hedman Lina, Manley David, Coulter Rory, and Östh John 2014 “Intergenerational Transmission of Neighbourhood Poverty: An Analysis of Neighbourhood Histories of Individuals.” *Transactions of the Institute of British Geographers* 39 (3): 402–417. doi:10.1111/tran.12040. 26074624 PMC4459034

[CIT0072] Wacquant Loïc. 2009 *Punishing the Poor: The Neoliberal Government of Social Insecurity. 1st ed. Politics, History, and Culture*. Durham, NC: Duke University Press.

[CIT0073] Wadsworth Martha E. 2012 “Working with Low-Income Families: Lessons Learned From Basic and Applied Research on Coping with Poverty-Related Stress.” *Journal of Contemporary Psychotherapy* 42 (1): 17–25. doi:10.1007/s10879-011-9192-2.

[CIT0074] Wakefield Sarah E.L., and Baxter Jamie 2010 “Linking Health Inequality and Environmental Justice: Articulating a Precautionary Framework for Research and Action.” *Environmental Justice* 3 (3): 95–102. doi:10.1089/env.2009.0044.

[CIT0075] Wolff Jonathan, and de-Shalit Avner 2007 *Disadvantage*. 1st ed Oxford Political Theory Oxford: Oxford University Press.

[CIT0076] Yoshikawa Hirokazu, Lawrence Aber J., and Beardslee William R. 2012 “The Effects of Poverty on the Mental, Emotional, and Behavioral Health of Children and Youth: Implications for Prevention.” *American Psychologist* 67 (4): 272–284. doi:10.1037/a0028015. 22583341

[CIT0077] Young Iris Marion. 2011 *Responsibility for Justice*. 1st ed Oxford Political Philosophy Oxford: Oxford University Press.

[CIT0078] Zimmerman Gregory M., and Messner Steven F. 2011 “Neighborhood Context and Nonlinear Peer Effects on Adolescent Violent Crime: Peer Violence and Neighborhoods.” *Criminology; An interdisciplinary Journal* 49 (3): 873–903. doi:10.1111/j.1745-9125.2011.00237.x.

